# Factors affecting mechanical (nociceptive) thresholds in piglets

**DOI:** 10.1111/j.1467-2995.2012.00737.x

**Published:** 2012-11

**Authors:** Andrew M Janczak, Birgit Ranheim, Torunn K Fosse, Sophie Hild, Janicke Nordgreen, Randi O Moe, Adroaldo J Zanella

**Affiliations:** 1 Department of Production Animal Clinical Sciences, The Norwegian School of Veterinary ScienceOslo, Norway; 2 Department of Pharmacology and Toxicology, The Norwegian School of Veterinary ScienceOslo, Norway

**Keywords:** nociception, pig

## Abstract

**Objective:**

To evaluate the stability and repeatability of measures of mechanical (nociceptive) thresholds in piglets and to examine potentially confounding factors when using a hand held algometer.

**Study design:**

Descriptive, prospective cohort.

**Animals:**

Forty-four piglets from four litters, weighing 4.6 ± 1.0 kg (mean ± SD) at 2 weeks of age.

**Methods:**

Mechanical thresholds were measured twice on each of 2 days during the first and second week of life. Data were analyzed using a repeated measures design to test the effects of behavior prior to testing, sex, week, day within week, and repetition within day. The effect of body weight and the interaction between piglet weight and behaviour were also tested. Piglet was entered into the model as a random effect as an additional test of repeatability. The effect of repeated testing was used to test the stability of measures. Pearson correlations between repeated measures were used to test the repeatability of measures. Variance component analysis was used to describe the variability in the data.

**Results:**

Variance component analysis indicated that piglet explained only 17% of the variance in the data. All variables in the model (behaviour prior to testing, sex, week, day within week, repetition within day, body weight, the interaction between body weight and behaviour, piglet identity) except sex had a significant effect (*p* < 0.04 for all). Correlations between repeated measures increased from the first to the second week.

**Conclusions and Clinical relevance:**

Repeatability was acceptable only during the second week of testing and measures changed with repeated testing and increased with increasing piglet weight, indicating that time (age) and animal body weight should be taken into account when measuring mechanical (nociceptive) thresholds in piglets. Mechanical (nociceptive) thresholds can be used both for testing the efficacy of anaesthetics and analgesics, and for assessing hyperalgesia in chronic pain states in research and clinical settings.

## Introduction

Piglets (*Sus scrofa*) may suffer from pain associated with different routine husbandry procedures such as tail docking and castration. They may also be subject to pain caused by mechanical damage such as crushing by the sow, butting and biting by litter mates and by diseases such as infectious arthritis. The ability to assess sensitivity to nociceptive stimulation in piglets is important for several reasons. The measurement of pain threshold can be used to assess the efficacy of anaesthetic and analgesic protocols, to monitor the effectiveness of different treatments on pain, and to map the degree of wound hyperalgesia after surgery (Slingsby et al. 2001). In addition to this, measures of nociceptive threshold can be used to describe inter-individual and inter-breed differences in nociceptive sensitivity as has been investigated for rodents (Mogil 1999) and humans (Nielsen et al. 2005).

One approach to assessing sensitivity to noxious stimulation in humans is to measure the threshold at which a subject responds to blunt force applied to the body using an algometer (Fischer 1987; Potter et al. 2006). A hand-held algometer has the advantage that it can be used for testing of mechanical thresholds at different areas of the body and proximity to wound or inflammatory sites. This methodology has been used previously for assessing pain related responses in piglets (Fosse et al. 2011), horses (Haussler & Erb 2006a,b; Haussler et al. 2007), sheep (Stubsjøen et al. 2009; Stubsjoen et al. 2010) and humans (Treede et al. 2002; Rolke et al. 2006). However, as a step in validating a model of pain sensitivity in piglets, assessment of repeatability and stability of the measure is essential. It has recently been shown that mechanical thresholds measured in young pigs are sensitive to a kaolin-induced inflammation and treatment with nonsteroidal anti-inflammatory drugs (Fosse et al. 2011). Although the thresholds obtained by using manually operated algometers have been shown to be reliable in humans (Potter et al. 2006) and horses (Haussler & Erb 2006b) their reliability has not been assessed for piglets. Sandercock et al. (2009) recently validated an automated device for pressure stimulation and testing of young pigs.

In this experiment we used a hand-held algometer for the measurement of mechanical (nociceptive) thresholds in piglets. The aim of the study was to describe the repeatability and stability of these measures, and to describe the effects of potentially confounding variables. Repeatability was assessed by calculating correlations between repeated measures recorded on the same test day. Stability was investigated by testing for changes in mechanical thresholds over repetition, day and week. In addition to this, we tested the effects of potentially confounding variables including piglet body weight, repeated testing, piglet sex and behaviour prior to testing on mechanical thresholds, as well as describing the variance in the data that could be ascribed to each of these factors.

## Material and methods

This experiment was performed with the permission of the animal experiments committee of the Department of Animal and Aquacultural Sciences, Norwegian University of Life Sciences (approved by the Norwegian Government) under reference number 832, based on a cost-benefit analysis. Sick animals were excluded from the experiment.

### Animals

The animals used for mechanical threshold testing (*n* = 44 Landrace × Yorkshire piglets) were selected randomly from four different litters (litter one: five males and eight females, litter two: four males and six females, litter three: five males and six females, litter four: seven males and three females) from the same room at an experimental farm. Sows were moved from the pregnant sow section to individual farrowing pens three to seven days before expected farrowing, and were loose housed. The farrowing pens were all of the same type (length × width: 3.30 m × 1.80 m), with concrete floors on the lying area, and a plastic coated slatted floor in the dunging area in the rear end of the pen (1.17 m × 1.80 m). The pens were cleaned, and fresh straw bedding material was provided every morning. The piglet creep area was located in one of the front corners of the pen, and had a solid, concrete floor covered with a thick layer of sawdust. This area was covered by a solid roof with a curtain to reduce air flow around the infrared heat lamp placed in the middle of the roof of the creep area. A sow feeder was placed in the opposite front corner of the pen. Artificial lighting was provided from 07:30 to 15:00 hours in addition to natural light from the windows.

The sows and the piglets had free access to water from two nipple drinkers. The sows were fed a standard concentrate diet twice daily. By the time of farrowing, sows were given four kg of concentrate, and this was raised by 0.5 kg per day until they reached an upper limit of eight to ten kg per day. Each sow was given a large amount of straw (around 2 kg) in the pen for nest building, and a thick layer of sawdust was put into the piglet creep area on the day before expected farrowing. All piglets were ear marked with a tattoo on the day of birth and given an intra muscular iron injection at three days of age. Piglets were provided with concentrate feed in the creep area.

### Mechanical (nociceptive) threshold testing

For data collection one animal was pseudo-randomly chosen (the choice was not based on any special criteria) within each of litters one to four. The piglet’s behaviour was registered before it was taken out of the pen for testing, as either passive (lying down) or active (not lying down). When one animal from each pen had been tested, this procedure was repeated until all animals in all four litters had been tested. After testing each piglet was colour-marked to prevent it from being accidentally used again. This procedure was followed in order to avoid the additional handling of animals prior to testing that would have been necessary in order to check ear tattoos (which were applied on the day of birth) for strictly randomized testing. Animals were weighed after testing one time each week. The piglets were carried gently to the test room by the same handler on all test days. The distance to the test room was 15–35 m, and the transportation procedure took maximum 1 minute. The piglets were then placed in a hammock with holes through which the legs hung in a steady position. The fixation apparatus made it possible for a single person, who was the same throughout the study, to hold and test the piglet without assistance. The separate room used for testing measured 350 × 350 cm. It was visually and acoustically isolated. It was heated to 30 °C to prevent cooling of piglets during testing.

The device used to measure the mechanical threshold was designed for measuring mechanical thresholds and mechanical tolerance in humans (Commander Algometer, JTECH Medical, UT). It consisted of a flat-tipped circular pin with a 0.2 cm diameter. We constructed this 0.2 cm diameter tip (0.031 cm^2^) for the present study because the smallest commercially available tip, which measured 0.5 cm^2^, did not induce withdrawal responses in all piglets at the cutoff force of 25 Newtons (N). This custom-made tip exerts a pressure of 8065 kPa at 25 N force, compared to the smallest commercially available tip which exerts a pressure of 500 kPa at 25 N force. For testing, the tip was pressed at a 90° angle against the back of the metacarpus/metatarsus of the piglets’ legs (supradigital palmar/plantar region) at a point that was predefined by marking a spot of the same diameter with a marker. Piglets that did not show a response before the cutoff was reached were assigned a measure of 25. During mechanical threshold testing, stimulation was always stopped as soon as the animal clearly attempted to withdraw its leg from the source of stimulation unless this movement was made when no force was applied. In cases where animals showed spontaneous flinching prior to application of force the worker waited for the animal to stop moving. No bruising or skin changes were visible as a result of mechanical threshold testing.

**Figure 1:**
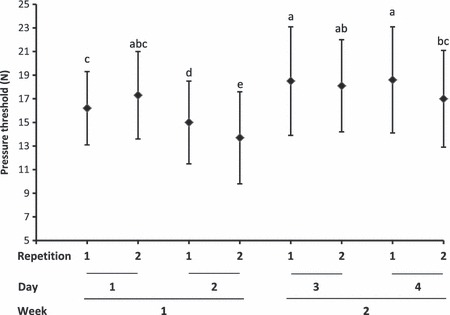
Plot showing mean ± SD of mechanical threshold measurements in 44 piglets, measured in Newtons, over the first week (repetition 1–4) and the second week (repetition 5–8). Two measurements were performed on each of the two test days each week. Values with different letters denote means for which corresponding LsMeans are significantly different (*p* < 0.05).

All animals were tested for their mechanical threshold in two repetitions on each of two different days during the first week of age, and again during the second week of age, giving a total of eight measurements per animal (Fig. 1). Each leg of the animal was stimulated once, by delivering a standardized, steadily-increasing force over a ten second period. Measurements were then repeated immediately for the same animal to produce a total of two measurements per leg. Each of the four legs were stimulated according to a Latin square design, i.e. legs were numbered one to four and tested in the same order for all animals, but the first leg to be tested changed for each group of four piglets (one from each litter). For each test day, there was therefore two data points for each leg. The measurements on different legs were averaged and used as a single measure giving two measurements of mechanical threshold for each piglet per test day. This was done because there were no differences between legs, or between front and back legs.

### Statistical analysis

The data conformed to the assumptions of the general linear model (normal distribution of residuals, equality of variance and linearity) and analyses were therefore performed using untransformed data. To analyze the effects of the different factors on mechanical thresholds, we used a mixed model analysis of variance (model 1) based on restricted maximum likelihood estimation with a repeated measures design, with behaviour prior to testing (active or passive), sex (male or female), week (1 or 2), day within week (1–2), repetition within day during the same week as class variables. The weight of piglet was included as a continuous variable. The interaction between weight and behaviour prior to testing was included in the model. Piglet was entered into model 1 as a random effect (Hatcher & Stepanski 1994). We tested effect of week, day, and repetition in order to evaluate the degree to which measures were stable over time. We tested the effects of behaviour prior to testing, sex and piglet weight in order to assess the effect of potentially confounding variables. The *t*-test was used to describe the stability of the measures. Results for mechanical thresholds are presented as LsMean ± standard error (LsMean ± SE) in the results text, because these are the values that are compared in the statistical analysis, whereas litter size, piglet weight, mean mechanical thresholds in the figure, and overall mean mechanical threshold are presented as mean ± standard deviation (mean ± SD) to present the uncorrected values for the data.

Another model (model 2) defining all effects as random was used to calculate the percentage of variance in mechanical thresholds that could be attributed to each variable. Pearson correlations between repeated measures were used to test the test-retest reliability (repeatability) of measures. Data for mechanical thresholds are presented in Newtons. Statistical tests were all two-tailed with a significance level of five %. Analysis was performed using SAS version 9.1 and JMP version 7.0.1 (SAS Institute Inc., NC, USA).

## Results

### General background information

The litter size (*n* = 4) was 11 ± 1.41 (mean ± SD), the weight of animals (*n* = 44) at 7 days of age 2.6 ± 0.6 kg and their weight at 14 days of age 4.6 ± 1.0 kg.

The overall mean mechanical threshold was 16.8 ± 4.2 N (mean ± SD). The results for individual legs showed that only in 1.5% of tests did piglets not withdraw their foot before the cutoff threshold of 25 N was reached. The cutoff threshold was never obtained on more than two feet for any individual piglet. Cutoff threshold values were obtained for two feet in three piglets. Cutoff threshold readings for one or more feet were obtained for a total of 13 different piglets.

### Stability of measures

Week of testing (*p* < 0.001), test day within week (*p* < 0.001), and repetition within day within week (*p* < 0.003) all affected mechanical thresholds (Model 1). Mechanical thresholds (all as LsMean ± SE) were higher the second week of testing = 18.0 ± 0.4 N) than the first week of testing (15.7 ± 0.4 N; *p* < 0.05; Fig. 1). Mechanical thresholds decreased over test days the first week (day 1: 16.9 ± 0.5 N, day 2: 14.4 ± 0.5 N; *p* < 0.05), but not the second week of testing (day 1: 18.2 ± 0.5 N, day 2: 17.7 ± 0.5 N; *p* > 0.05). Repeated measurements were more stable on the second week of testing than on the first (Fig. 1).

### Repeatability of measures within day as indicated by test-retest reliability

The Pearson correlations between repeated measures for the same test day were *r* = 0.46 for day 1, *r* = 0.60 for day 2, *r* = 0.56 for day 3 and *r* = 0.79 for day 4.

### Effect of potentially confounding variables

There was a significant interaction between piglet weight and behaviour prior to testing (*p* < 0.03), and behaviour prior to testing tended to affect the mechanical threshold (*p* < 0.07; Table 1). Heavier piglets had higher mechanical thresholds (regression coefficient = 3.0; SE = 0.63; df = 40.11; *t* = 4.74; *p* < 0.0001), and active behaviour prior to testing interacted with body weight to further increase the mechanical threshold (regression coefficient = 0.69; SE = 0.32; df = 321.2; *t* = 2.18; *p* < 0.03). Sex had no effect on mechanical thresholds (*p* = 0.5).

**Table 1:** Model 1: fixed effect tests for analysis of variance

Source	DF Numerator	DF Denominator	*F* Ratio	*p* > *F*
Behaviour (active or passive)	1	326.4	3.41	0.07
Week (1 or 2)	1	295.4	51.40	<0.0001
Day within week	2	292.8	17.15	<0.0001
Repetition within day within week	4	292.2	4.17	0.003
Body weight	1	40.11	22.46	<0.0001
Body weight × behaviour	1	321.2	4.75	0.03
Sex	1	39.98	0.45	0.5

The variance component analysis (Table 2) indicated that the model as a whole explained 67.40% of the variation in the data whereas 32.60% was residual variance. Piglet contributed to only 17.00% of the total variance. The remaining variance could be attributed to body weight (32.47%), week in which measurements were made (7.58%), test day within week (4.93%), repetition within day in the same week (2.40%) and the interaction between body weight and behaviour prior to testing (2.79%).

**Table 2:** Model 2: variance component estimates. The variance ratio is the variance component divided by the residual variance. The variance component estimate is the relative contribution of each factor in explaining the variance in the dependent variable. The 95% lower and 95% upper are the lower and upper limits of the 95% confidence interval for the variance components

Random effect	Variance ratio	Variance component estimate	Standard Error	95% lower	95% upper	% total
Behaviour (B)	0.02	0.20	0.37	−0.53	0.92	1
Piglet	0.52	4.31	1.20	1.96	6.66	17
Week (W)	0.23	1.92	3.98	−5.87	9.71	8
Day within W	0.15	1.25	1.68	−2.04	4.53	5
Repetition within day within W	0.07	0.61	0.57	−0.50	1.72	2
Weight	1.00	8.23	12.72	−16.71	33.16	32
Weight × B	0.09	0.71	1.28	−1.80	3.21	3
Sex	−0.02	−0.14	0.17	−0.47	0.20	−1
Residual		8.26	0.68	7.07	9.78	33

B, behaviour and W, week.

## Discussion

This study describes factors that influence nociceptive mechanical thresholds recorded with a handheld algometer, and the stability and repeatability of these measures. The results indicate that several factors, including the timing of testing, and the weight of piglets that are tested, influence mechanical threshold recordings. Furthermore, high correlations were found only for responses to stimulation after several days of habituation to the test procedure. Apart from the present study, Fosse et al. (2011) have previously used similar equipment for quantifying analgesic effects of different drugs in piglets. Their study suggests that this methodology has internal validity for measuring nociceptive sensitivity, but they do not describe the influence of potential confounding factors or the repeatability of measures. Sandercock et al. (2009) validated a mechanical pressure application and measurement device for use in young pigs, but the equipment used was highly automated. The present study thus provides novel information regarding the stability and repeatability of measures when using a simpler handheld algometer for mechanical threshold testing in piglets. Measurement of mechanical thresholds has previously been used in sheep (Welsh & Nolan 1995; Stubsjøen et al. 2009; Stubsjoen et al. 2010), cattle (Ley et al. 1996), and horses (Haussler & Erb 2006b). These studies support the suggestion of Viñuela-Fernández et al. (2007) that this methodology may be useful for quantifying the efficacy of anesthetics and analgesics and assessing hyperalgesia in chronic pain states in research and clinical settings. Although the repeatability of handheld algometers may not be as high as automated stimulation and force measurement devices of the type described by Sandercock et al. (2009), they are cheap and relatively simple to use.

Mechanical thresholds were measured repeatedly in 44 piglets twice on two test days the first week of life, and twice again on two different test days the second week of life. The recorded mechanical thresholds decreased over test days during the first week but were more stable during the second week, with a significant decrease only for the last measurement during the second week. There was also an increase in mechanical thresholds from the first to the second week, possibly associated with the increasing body weight of piglets, which accounted for a large percentage of the variance in the data and had a positive influence on mechanical thresholds. The variability in measurements as indicated by the standard deviation varied little between repetitions. These results suggest that although the absolute level of measurement may increase with increasing age, experience (habituation), and weight of piglets, measurements become more stable as the animals grow or habituate to the test procedures. The interpretation that measurement stability increases with repeated testing, body weight or animal age is also supported by the observation that correlations between repeated measures for the same test day increased from 0.5 to 0.8 from the two first repetitions to the two last two repetitions. This particular finding corresponds well to the study by Stubsjoen et al. (2010), who also documented an increasing correlation over time between repeated measures for mechanical thresholds in sheep. Based on the present experimental design, it is not possible to know whether the increased stability in measurement values was caused by experience, age, increasing body weight, or a combination of these factors. Furthermore, the human performing the measurements may have become more precise over time and this could also contribute to the higher repeatability of the last repetitions. The experiment aimed at applying a force that increased constantly from 0 to 25 N over a period of 10 seconds. Irregularities in the rate of increase in force potentially could cause a mismatch between repeated measures of mechanical thresholds resulting in low repeatability. Although this cannot be quantified based on the data in the present study, the possibility that the investigator became better at standardizing stimulation with increased experience cannot be excluded. In conclusion, it is clear that there is not a high level of stability or repeatability for mechanical thresholds measured in naïve piglets at 1 week of age, but that stability and repeatability increases to acceptable levels with repeated testing of the same animals during a second week of testing.

This study presents the commonly used Pearson correlation coefficient as a measure of test-retest reliability to allow comparison with other studies. Cronbach’s alpha is inappropriate as it is intended for assessing internal consistency between different measures (items) thought to reflect the same construct (Cortina 1993). In the present case Cronbach’s alpha therefore over-estimates consistency between repeated measurements recorded on the same day (0.63 for day 1, 0.75 for day 2, 0.71 for day 3 and 0.88 for day 4) compared to the Pearson correlations (0.46 for day 1, 0.60 for day 2, 0.56 for day 3 and 0.79 for day 4). The ICC is an alternative statistic that quantifies test-retest reliability for the same repeated measure (McGraw & Wong 1996). The ICC can be calculated on the basis of the variance component analysis used in the present study after adding the interaction between piglet and repetition within test day for data sorted by week and day. The ICC for each test day is then calculated as the ratio of the variance component estimate (var) for piglet to the sum of the variance component estimates for piglet, repetition within test day, interaction between piglet and repetition within test day, and residual variance [ICC = piglet_var_/(piglet_var_ + repetition_var_ + piglet_var_ × repetition_var_ + residual_var_]. For the present data the ICC was 0.29, 0.40, 0.52 and 0.65 between repeated measures on days 1-4, respectively. The results and conclusions are thus similar to what one finds when using Pearson correlation coefficients, indicating that test-retest reliability increases over time. The ICC does, however, in agreement with a previous report by Miller et al. (2005) result in a lower estimate of test-retest reliability. This is due to the fact that it is calculated after removing the effects of confounding variables such as body weight, behaviour prior to testing and sex.

The observation that the mechanical thresholds sank from the first to the second day of testing during the first week suggests that piglets become more sensitive with repeated testing over successive days, or that their ability to respond to stimulation improves with time. The latter could possibly be due to CNS development. In human preterm infants pain perception is present, but the ability to show a pain response improves with age (Ranger et al. 2007). A similar reduction in mechanical threshold was observed for the second repetition on the second day of the second week. The stress caused by handling and isolation involved in testing the animals (Jensen et al. 1995a) potentially could have led to stress-induced analgesia (Hayes et al. 1978). Although this initially would cause elevated mechanical threshold readings, it could also potentially later lead to a reduction of mechanical thresholds over time due to habituation to the isolation and handling procedures and a concurrent reduction in stress with repeated testing. In addition to this, the change in mechanical threshold over time may also have been due to maturation of the CNS and corresponding centrally mediated cognitive processes, whereby the animals’ ability to respond to, and thus terminate stimulation by showing the appropriate response, improved with repeated testing.

The combination of the different variables included in the variance component analysis explained 67% of the variance in the data. Seventeen per cent of this variance could be attributed to differences between piglets, and 33% could be attributed to piglet body weight. It is therefore imperative that studies using mechanical thresholds in young pigs either standardize or otherwise take into account the body weight of animals. The variability attributed to piglet identity can be viewed as reflecting a stable individual trait (see Jensen et al. 1995b; Spoolder et al. 1996; Ruis et al. 2000; Micalos et al. 2009) related to sensitivity to mechanical stimulation. Remaining variability could be attributed to the time-related variables discussed above (15%), and the interaction between body weight and behaviour prior to testing (2.8%). Piglets that were active prior to testing tended to be less sensitive, as indicated by higher mechanical thresholds during testing, although variation in behaviour prior to testing explained under one % of the variation in the data.

This study describes the use of mechanical threshold measurement in young piglets at one and 2 weeks of age weighing 2.6 ± 0.6 kg (mean ± SD) and 4.6 ± 1.0 kg, respectively. Piglets weighing about 5.5 kg were also used in the study by Fosse et al. (2011). Sandercock et al. (2009) used 30 day old piglets weighing about 8–10 kg, indicating that this methodology should also be useful for testing mechanical thresholds in weaned pigs. Control piglets in this study had a mean mechanical threshold of 8.4 N measured on the foot pad, which is considerably lower than the 17 N recorded when force was applied to the metacarpus/metatarsus in the present study. These results suggest that manual stimulation and mechanical threshold measurement using an algometer should be a viable method in pigs up to 10 kg. However, pigs weighing 80–100 kg do not show a response when using a cut-off of 30 Newtons (pers. comm.). It appears that stress-induced analgesia caused by handling, isolation and fixation of larger pigs may greatly limit the applicability of this methodology unless long periods of habituation are used. Furthermore, the effort and/or technical requirements for fixating such large animals are considerable.

## Conclusions

This study indicated that measurements of mechanical thresholds in piglets using a manually operated algometer had acceptable repeatability after habituation to the test procedure during the second week of testing. Mechanical thresholds changed with repeated testing and increased with increasing body weight, indicating that temporal variables and animal body weight should be taken into account when measuring mechanical (nociceptive) thresholds in piglets.
